# Mechanisms of Viral Degradation of Cellular Signal Transducer and Activator of Transcription 2

**DOI:** 10.3390/ijms23010489

**Published:** 2022-01-01

**Authors:** Sailen Barik

**Affiliations:** EonBio, 3780 Pelham Drive, Mobile, AL 36619, USA; eonbiohelp@gmail.com

**Keywords:** RNA virus, interferon, STAT, immunity, proteasome, ubiquitin, nonstructural protein

## Abstract

Virus infection of eukaryotes triggers cellular innate immune response, a major arm of which is the type I interferon (IFN) family of cytokines. Binding of IFN to cell surface receptors triggers a signaling cascade in which the signal transducer and activator of transcription 2 (STAT2) plays a key role, ultimately leading to an antiviral state of the cell. In retaliation, many viruses counteract the immune response, often by the destruction and/or inactivation of STAT2, promoted by specific viral proteins that do not possess protease activities of their own. This review offers a summary of viral mechanisms of STAT2 subversion with emphasis on degradation. Some viruses also destroy STAT1, another major member of the STAT family, but most viruses are selective in targeting either STAT2 or STAT1. Interestingly, degradation of STAT2 by a few viruses requires the presence of both STAT proteins. Available evidence suggests a mechanism in which multiple sites and domains of STAT2 are required for engagement and degradation by a multi-subunit degradative complex, comprising viral and cellular proteins, including the ubiquitin–proteasomal system. However, the exact molecular nature of this complex and the alternative degradation mechanisms remain largely unknown, as critically presented here with prospective directions of future study.

## 1. Introduction

Innate immunity, represented by type I interferon (IFN) and typified by IFN-alpha and IFN-beta (IFN-α and -β), forms the core of the cellular antiviral defense system. Ubiquitously present in all vertebrate cells, the IFN genes are transcriptionally induced in response to a variety of foreign molecules, such as double-stranded viral RNA in the cytoplasm. The secreted IFN acts as a warning signal and is recognized by IFN receptors on the surface of the neighboring cells, which elicits a signaling cascade via the activation of Janus kinases (JAKs) [1]. Activated JAK phosphorylates specific tyrosine residues in multiple members of the signal transducer and activator of transcription (STAT) family [2,3]. Phosphorylation of STAT1 and STAT2 allows association and recruitment of the interferon regulatory factor 9 (IRF9), resulting in the formation of the IFN-stimulated gene factor 3 (ISGF3) [4,5,6]. The heterotrimeric ISG3 complex translocates to the nucleus, where it binds the IFN-response elements (ISRE) in the promoters of a large number of interferon-stimulated genes (ISGs), leading to the transcriptional induction of the latter [7]. Since many ISGs, if not all, possess antiviral properties, this leads to an antiviral state of the cell [8,9,10]. In response, several viruses have evolved mechanisms that suppress diverse steps of the IFN induction and response pathways, of which suppression of the STATs is one. Inhibition of phosphorylation or promotion of degradation of the STAT proteins relieves the block on virus replication, allowing optimal growth of the virus and the resultant morbidity and mortality. Viral suppression of the STATs, therefore, is an important area of host–virus interaction, for both scientific and clinical reasons.

Although viruses affect multiple factors in the IFN pathway, this review is primarily aimed at STAT2 degradation, for reasons elaborated here and in order to maintain focus and depth. The STAT pathway of IFN induction is relatively ancient and evolutionarily conserved, hence operational in essentially all living cells and tissues. The STAT family members are divergent in sequence, as seen among the seven paralogs in the mammalian STAT family (STATs 1, 2, 3, 4, 5a, 5b, 6) [11,12,13,14,15]. The divergence of STAT2 among multiple species is particularly prominent in the C-terminal transactivation domain (TAD). Nonetheless, for its particularly important role in type I IFN signaling, STAT2 and its destruction by specific viruses has received substantial attention. First of all, although the IFN response pathway leads to robust induction of the large family IFN-stimulated genes (ISGs), the preformed STAT2–IRF9 complex allows basal low-level expression of roughly a quarter of the ISGs [12,16,17,18]. Indeed, relatively recent studies have revealed that unphosphorylated STAT2 and STAT1 (U-STAT2, U-STAT1), lacking the IFN-triggered phosphorylation, can combine with IRF9 to form the unphosphorylated version of ISGF3 (U-ISGF3) [17,19,20]. The cardinal role of STAT2 in ISG induction was further established in several other studies. In one study, STAT1-deficient cells that overexpress STAT2 supported IFNα-induced expression of key antiviral ISGs and inhibited the growth of encephalomyocarditis virus (EMCV) and vesicular stomatitis virus (VSV) [16]. Early studies also showed that in some cases, type I IFN can promote STAT1-independent signaling that is still STAT2-dependent [21]. A similar conclusion was reached in another study that identified a set of IFN-inducible genes whose expression was ISGF3-indepenedent but dependent on the DNA-binding domain of STAT2 [22]. The STAT2–IRF9 complex, devoid of STAT1, also plays an important role in the signaling by type II IFN—namely, IFNγ [18,23,24,25].

Analyses of STAT2 knockout (KO) animals and naturally occurring mutations in humans have lent further credence to the paramount importance of STAT2 in antiviral immunity. Such reports include but are not limited to the early observation of enhanced VSV virulence in KO (STAT2^–/–^) mice [26], increased pathogenesis of Rift Valley fever virus [27] and Crimean-Congo hemorrhagic fever virus [28], and increased dissemination of SARS-CoV-2 in STAT2 KO hamsters [29]. In human subjects, heterozygous carriers of STAT2 null mutations are clinically unaffected; however, those with complete loss of STAT2 function showed the interesting phenotype of severe viral disease from live-attenuated virus vaccines, such as those for measles, mumps, and rubella (MMR) and varicella zoster virus [30,31]. Finally, for many viruses, STAT2 of the host has been shown to determine host specificity of the virus, such that these viruses cause disease in humans only, as detailed later for individual viruses.

Considering the multifaceted and singular abilities of STAT2, it stands to reason that a multitude of viruses target STAT2 to ensure a stringent suppression of the IFN response. Moreover, the predominant mechanism used by the viruses is to fully degrade STAT2 by the ubiquitin–proteasome system (UPS) described below. Degradation causes irreversible loss of STAT2, which is otherwise a relatively stable protein, with a half-life exceeding 24 h [32]; moreover, as the UPS degrades both the phosphorylated and the unphosphorylated forms of STAT2 (U-STAT2), it ensures suppression of all forms of STAT2 function regardless of the extent of phosphorylation. Of note, much of the analyses of STAT2 degradation by viral proteins, including biochemical as well as structural studies, were conducted with transiently expressed recombinant STAT2 (that is essentially U-STAT2) and recombinant viral proteins. This is also true for viruses that target STAT1 for degradation; for example, SV5 infection in cell culture does not require that the cells be pre-treated with IFN for the STAT1 degradation to occur [33,34]. Despite this importance of STAT2 in host–virus interaction, studies of the mechanism of its degradation have been fragmentary, often lacking molecular details. Here, a mechanistic view of STAT2 degradation is presented, based on available reports from a variety of viruses (Table 1), with emphasis on protein–protein interactions at the molecular level.

## 2. STAT2 Degradation by the Ubiquitin–Proteasome System (UPS)

### 2.1. Basic Features of the Ubiquitin–Proteasome System

Proteasomal degradation of ubiquitinylated protein substrates is a major mechanism of target degradation of proteins, which will be briefly described here. The various facets of the ubiquitin–proteasome system (UPS), as it is called, have been covered in several comprehensive reviews [35,36,37,38]. Traditionally, the first tier of evidence for proteasomal degradation of a substrate is derived from the protection of the substrate by specific proteasome inhibitors—namely, MG132 (Cbz-leu-leu-leucinal) and lactacystin [39,40]. Needless to say, this evidence was obtained in all examples reviewed here. In UPS, substrate proteins are first conjugated to ubiquitin (Ub), a highly conserved 76-amino acid polypeptide that covalently attaches to a lysine residue of the target protein via the invariant glycine residue at its C-terminus. The process, known as “ubiquitylation”, is catalyzed by the sequential action of three dedicated enzymes: ubiquitin-activating enzyme (E1), ubiquitin-conjugating enzyme (E2), and ubiquitin ligase (E3). First in this sequence, the E1 enzyme produces a ubiquitin–adenylate intermediate, from which the Ub is transferred to E2 and finally to the target protein by the participation of E3. The number of these enzymes in the cell increase almost exponentially; the human cell, for example, contains only two E1 enzymes, approximately 40 E2s, and an estimated 650–1000 E3s [33,34,35,36,37,38,39,40,41,42,43]. The exact number of E3 enzymes remains unknown due to their diversity and large, multisubunit nature. The incremental number of E1 < E2 < E3 reflects the fact that these enzymes are increasingly dedicated to individual substrates and that the UPS processes an enormous number of proteins, likely several thousands. Predictably, one E3 ligase can ubiquitylate multiple substrates. Since Ub itself contains seven lysines, one Ub molecule can attach to multiple other Ubs in various arrangements, leading to polyubiquitin chains of diverse lengths. As a rule, substrates that are conjugated via Lys48 of Ub are targeted for degradation, while conjugation via the Lys63 of Ub promotes protein–protein interaction. The K48-ubiquitin tags are recognized by the 26S proteasome, which engages the protein at a disordered region [44,45] and transports it into the proteolytic core while unfolding the secondary structure. The disordered region is likely a major code for the initiation of degradation, as there are examples where a ubiquitylated protein escaped degradation for the lack of a disordered region. The E2 enzyme UbcH10 (also named UBE2C), for example, requires ubiquitylation as well as its 29 aa long unstructured N-terminus [46]; in this mechanism, the unstructured region of UbcH10 directly bound the proteasome, and initiated substrate translocation, but degradation also required ubiquitylation. Several unstructured sequences can be seen in the STAT2 structure (See later), but it is not known which one of them, if any, is involved in STAT2 recognition by the proteasome. Examples also exist of non-ubiquitylated proteins that are degraded by the proteasome, as reviewed recently [47]. Such exceptions include: Rpn4, a transcriptional regulator of proteasome homeostasis; thymidylate synthase, an enzyme required for production of DNA precursors; and ornithine decarboxylase, the initial enzyme committed to polyamine biosynthesis. It is worth mentioning that such exceptions have not been encountered for STAT2 degradation; in other words, in all known examples of viral degradation of STAT2, ubiquitin conjugation was needed.

Finally, the proteasome digests its substrate proteins into small peptides that are likely further hydrolyzed into amino acids to become part of the cellular amino acid pool. The Ub moieties are cleaved off and reused [45].

### 2.2. The E3 Ligases: Assembly and Function

**Figure 1:**
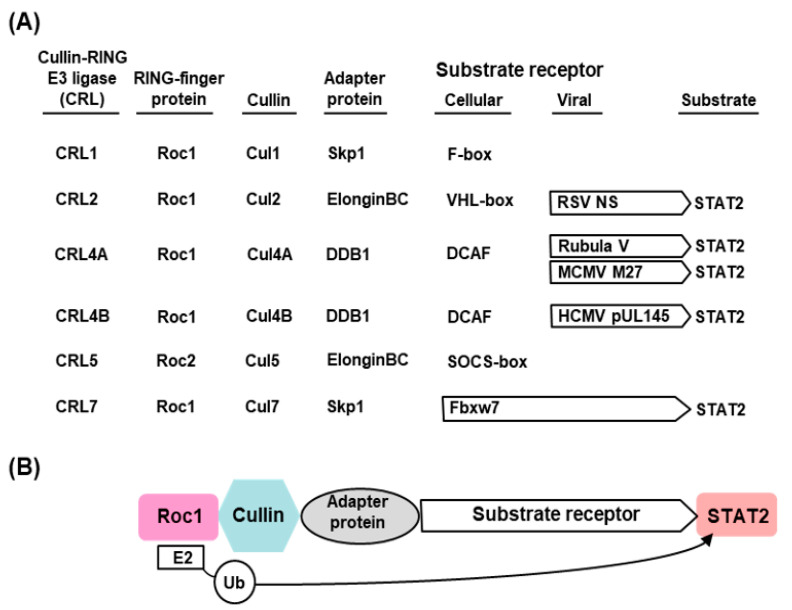
(**A**) Summary of different classes of Cullin–Roc E3 ligase (CRL) and representative viral proteins that exploit them for STAT2 degradation. (**B**) A general schematic of a CRL structure [59], presented in a linear fashion. In reality, this arrangement brings the Roc1-assciated E2 Ub-conjugating enzyme in close proximity to the substrate, resulting in the transfer of the Ub moiety from E2 to the substrate. Of the eight classes of CRLs (CRL1, 2, 3, 4A, 4B, 5, 7, and 9), only six that have been mentioned in this review are listed here. Each CRL is anchored by a distinct Cullin isoform and contains four core components: a Cullin (Cul) that acts as a scaffold, an E2 Ub-conjugation enzyme (Roc), a substrate receptor that recognizes the ubiquitination target, and an adaptor protein that links the substrate receptor to the Cullin. Only STAT2, which is the main focus of this article, is shown here as the substrate, along with the cognate CRLs that have been experimentally demonstrated to ubiquitylate it. The receptors that recognize STAT2 for ubiquitylation (and ultimate degradation by the proteasome) are marked with arrow-headed box. In the uninfected cell, cellular proteins (as listed) serve as receptors to ubiquitylate a variety of cellular proteins (not shown), whereas in the virus-infected cell specific viral proteins take their place.

The E3 ligases constitute a highly complex family of enzymes with enormous variety and several interacting proteins, and therefore, only a very brief description, relevant for STAT2 degradation, will be provided here. Interested readers may consult research papers and detailed reviews on the subject [38,41,48,49]. The known E3 ligases belong to four major families: RING-finger, HECT, U-box, and PHD-finger, of which the RING-finger is the most common and includes the SCF complex (Skp1–Cullin–F-box protein complex). The SCF complex is composed of four proteins: Rbx1, Cul1 (Cullin1), Skp1, and an F-box protein [49,50]. Cul1 also belongs to the Cullin family of large, helix-rich scaffold proteins [51,52], each of which forms specific E3 ligase complexes. Cul4 (Cullin4), for example, is a 759 aa long polypeptide that associates with DDB1 and Roc1 to form the DDB–Cul4–Roc1 ligase complex [51,52,53,54], important for histone methylation [53] and cellular DNA repair [31,48] and also for STAT degradation in paramyxovirus-infected cells (Section 2.3.2). Another subclass of E3 ligase complex that is important for STAT2 degradation by pneumoviral nonstructural proteins (Section 2.3.3) is the elongin C–Cullin–SOCS (ECS) box-type ligases that include the Von Hippel–Landau tumor suppressor protein and the suppressor of cytokine signaling (SOCS)-family proteins [51,55,56]. As described in detail, the Cul-RING E3 Ub ligases (CRLs) are the largest RING-type of E3 family consisting of approximately 240 members, whereby the E2-binding RING Ub ligases are Roc1/Roc2 (alternatively called RocA/B or Rbx1/2), which play an important role in viral STAT2 degradation (Figure 1) [57,58].

Together, these subunits and domains allow the ligase to function with high efficiency, coupled with selectivity. Notwithstanding the many major advances in our understanding of the UPS, how a protein is initially chosen for degradation and how the specificity of pairing between an E3 ligase and its Ub-conjugated protein substrate is achieved still remain largely unsolved mysteries. (More on this in appears in Section 4.1 and Section 4.2.)

### 2.3. Viruses and Viral Proteins That Promote STAT2 Degradation by the UPS

While the cellular protein degradation machinery, predominantly the UPS, catalyzes the actual degradation of STAT2, specific viral proteins (such as flaviviral NS5, paramyxoviral V, orthopneumoviral NS, M27 of mouse CMV, pUL145 of human CMV) initiate the process and make it substrate-specific; thus, the viral protein can be likened to the “conductor of the UPS orchestra”, who is also a direct participant. In molecular terms, the viral protein acts in the role of a scaffold subunit in the assembly of the UPS complex; for a subset of viruses, the viral protein itself has been shown to possess an E3 ligase activity, thus potentially obviating the need to recruit a cellular E3 ligase. The latter category includes the HSV2 (herpes simplex virus 2) protein, ICP22 (infected-cell protein 22), which is an E3 ubiquitin ligase and is responsible for ubiquitylation and degradation of all three subunits of ISGF3—namely, STAT1, STAT2, and IRF9 [60,61]—exerting a strong suppression of IFN response. Regardless of the two mechanisms, we have named such complexes “virally assembled degradation complex” or VADC, for ease of referral. Previously, the acronym VDC, standing for “V-dependent complex”, “virus-degradation complex”, or “V-DDB1-Cul4A”, was used to describe similar complexes assembled by the rubuloviral V proteins [62]. This section summarizes representative examples of diverse STAT2-suppressor proteins of virus families, listed in Table 1, and their molecular mechanisms.

#### 2.3.1. *Flaviviridae*

Flaviviruses constitute serious pathogens, transmitted mainly by mosquitoes, and cause debilitating infections in humans that are often lethal [63]. They are enveloped viruses with nonsegmented single-stranded positive sense RNA genome that is ~10 kb long. Prominent members include dengue virus (DENV), Japanese encephalitis virus (JEV), West Nile virus (WNV), yellow fever virus (YFV), and Zika virus (ZIKV) [63,64]. Inside the host cell cytoplasm, the flavivirus genome is translated into a single, large polyprotein that encodes three structural and seven nonstructural (NS) proteins (NS1, NS2a, NS2b, NS3, NS4a, NS4b, and NS5) [65]. Individual NS proteins are liberated by polyprotein cleavage, mediated initially by host proteases and then also by NS3, which is a protease. Largest among the non-structural proteins (~900 amino acids long in ~3400 amino acid polyprotein), NS5 functions as both an RNA-dependent RNA polymerase (RdRP), which is absolutely required for copying of the viral genomic RNA and as a methyltransferase enzyme that promotes 5′-capping of the viral mRNA, allowing its efficient translation. An amazingly multitasking protein [66,67], NS5 of flaviviruses also suppresses cellular IFN response [68]. A subset of NS5 (Table 1) promotes proteasomal degradation of STAT2, as shown in detail for the DENV and ZIKV orthologs [68,69,70,71,72,73,74,75,76,77,78]. Interestingly, NS5 of both viruses were found to exhibit STAT2 species specificity, i.e., they degraded human STAT2 (hSTAT2), but not mouse STAT2 (mSTAT2), as elaborated later. This was further confirmed by swapping recombinant STAT2 proteins between human and mouse cells, whereby the sensitivity to the virus followed hSTAT2 and resistance followed mSTAT2, regardless of the background of the cell [68,69,70]. To elaborate, normal human cells are ZIKV sensitive, while the mouse and murine cells are resistant; however, human cells become resistant if the native hSTAT2 is replaced by recombinant expression of mSTAT2. Reciprocally, knockdown of mSTAT2 in mouse cells highly stimulated ZINV replication. NS5, therefore, acts as an important determinant of host tropism for these viruses. It is important to note here that STAT2 plays a role in determining such host tropism in several other viruses as well, such as human metapneumovirus (HMPV), which can infect cells containing human STAT2 but not those expressing murine STAT2 [79]. The NS5 protein of YFV, which sequesters STAT2 instead of degrading it, also binds hSTAT2 and not mSTAT2 [80,81]. As described later, the sensitivity of hSTAT2 and resistance of mSTAT2 also extends to the NS proteins of RSV, a STAT2-degrader pneumovirus (Section 2.3.3). The overall picture that emerges is that the rodent STAT2 has evolved to be more resistant to viral suppression than its human counterpart. The divergent nature of the mouse STAT2 has been noted [82], which may shed light on the molecular mechanism of STAT2 degradation, but this remains to be fully exploited.

Dengue virus (DENV), another major flavivirus, causes the namesake dengue fever, dengue hemorrhagic fever, and dengue shock syndrome. Similar to ZIKV, the DENV NS5 degrades STAT2 through UPS, although the molecular details of the mechanism are not identical. Unlike its ZIKV counterpart, the DENV NS5 must be presented as the polyprotein precursor for STAT2 degradation to occur. In other words, whereas mature NS5 of ZIKV, recombinantly expressed, readily binds and degrades STAT2, that of DENV only binds but does not degrade STAT2 [68]. The simplest explanation is that the ability of NS5 to recruit the UPS to STAT2 requires a specific fold of the NS5 protein that is only attained in the context of the polyprotein. Evidently, resolution of the mechanism must await an analysis of the dynamic cotranslational change of the nascent polyprotein structure coupled with its interaction with the other components of the UPS, which is a major undertaking. Additionally, unlike that of ZIKV, the NS5 of DENV uses the E3 ligase UBR4 for STAT2 degradation and acts as an adapter between UBR4 and STAT2 [83], illustrating another layer of mechanistic difference.

Recently, the cryo-EM and crystal structures of hSTAT2 in complex with the NS5 of DENV and ZIKV) were solved [75], which revealed two-pronged interactions between the two proteins (Figure 1). The structure of the DENV NS5, complexed with UBR4, has not been determined, but is expected to provide interesting similarities and contrast between the two flaviviral NS5 functions. UBR4, an exceptionally large (~600 kDa) E3 ligase that belongs to the family of ligases that is involved in the degradation of N-end rule substrates, contains a common sequence eponymously called the “UBR motif” [84]. Several UBR proteins, including UBR4, also share a 70-amino acid Cys-rich, zinc finger-like domain, termed the UBR box. It will be interesting to know whether or how these domains function in DENV NS5 activity.

**Figure 2:**
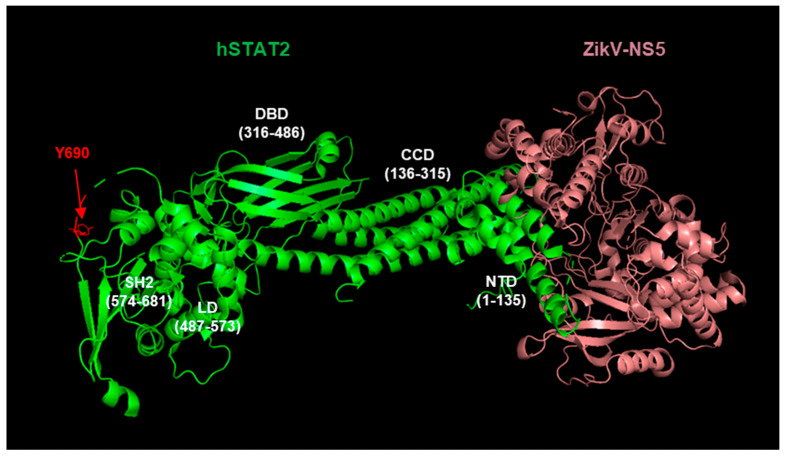
Cryo-EM structure (PDB 6WCZ) [75] of the complex of human STAT2 (green color) and ZIKV NS5 (salmon color). The various defined domains of STAT2 are indicated in white letters and residues numbers: N-terminal domain (NTD) that binds to the RdRP domain of NS5 (not labeled); coiled-coil domain (CCD) that binds within a cradle of the methyltransferase domain of NS5 (not labeled, behind NS5, away from viewer); DNA-binding domain (DBD), essential for the transcription function of STAT2; Src-homology domain 2 (SH2), involved in oligomerization with phospho-STATs, essential for the IFN pathway; the linker domain (LD) that connects the DBD to SH2 domain; lastly, the most abundantly phosphorylated site, Tyr690 (Y690, shown in red color), strategically located between the SH2 and the C-terminal transactivation domain.

Functional analysis, guided by the refined structures, showed that the methyltransferase and RdRP domains of NS5 form a cleft that cradles the coiled-coil domain of hSTAT2, preventing hSTAT2–IRF9 interaction; additionally, the NS5 RdRP domain binds the N-terminal domain of hSTAT2 (Figure 2). Disruption of these interactions abrogated NS5-mediated hSTAT2 degradation, interferon suppression, and viral infection. These results are also in agreement with previous demonstration that the difference in NS5-mediated binding and degradation between hSTAT2 (NS5-sensitive) and mSTAT2 (NS5-resistant) maps largely to the coiled-coil domain [85]. Very recently, the coiled-coil domain was also shown to be necessary and sufficient for STAT2 degradation by ZIKV NS5 [86], suggesting conservation of this mechanism between these two flavivurses. The structure of the full NS5 ubiquitin ligase complex for either virus is yet to be determined.

There are several important exceptions to NS5-induced STAT degradation [87] that include the NS5 of WNV, another flavivirus, which inhibits STAT1 phosphorylation but does not degrade it [88], even though the amino acid sequences of NS5 of WNV, DENV, and ZIKV are nearly 70% similar [73]. Thus, the mechanistic uniqueness of WNV NS5 is due to specific amino acid changes that remain unmapped. The three-dimensional structure of WNV NS5 or its complex with STAT1 has not been determined. Lastly, several flaviviral NS proteins other than NS5, such as ZIKV NS2A, also inhibit STAT2 through binding and sequestration [89]. These results demonstrate both the variety and the multiplicity of the flaviviral IFN suppression factors.

#### 2.3.2. Paramyxoviridae

##### Transcriptional RNA Editing of the Viral P Gene Produces Multiple Immune Suppressor Proteins

The *Paramyxoviridae* family belongs to the large group of viruses that contain a nonsegmented (alternatively called “unsegmented”) negative sense, linear, single-stranded RNA genome (belonging to the order *Mononegavirales*) [90]. As in any RNA virus (e.g., *Flaviviridae*, described above), they encode an RNA-dependent RNA polymerase since the animal host cell lacks such an activity. Being negative sense (i.e., anti-mRNA sense), the *Paramyxoviridae* genome cannot be translated as such but must first be transcribed into mRNA, which is then translated by the host cell translational machinery to produce de novo viral proteins, after which the viral replication escalates. Similar to the flaviviruses, many members of the *Paramyxoviridae* family (henceforth referred to as “paramyxovirus” for simplicity) have evolved specific viral genes that suppress type I IFN to promote virus growth and pathogenicity that are of significant health concern. A few examples are measles virus (MeV), Rinderpest virus (RPV), canine distemper virus (CDV), parainfluenza virus 2 (also called SV5), and human parainfluenza virus (hPIV) and mumps virus (MuV), of which the last two degrade STAT2 and are the most relevant for this article (Table 1).

**Figure 3:**
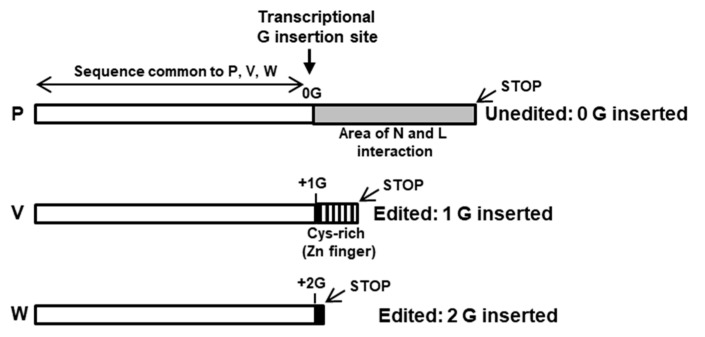
Co-transcriptional mRNA editing in *Paramyxoviridae* to generate multiple proteins from the P gene. A consensus schematic, modeled after Kato et al. [101], shows insertions of G nucleotides in the mRNA as well as the corresponding protein sequences and their relevant regions (boxed). Whereas the faithful copy of the gene (i.e., no G insertion, or 0G) produces the mRNA that translates into the longest protein, the phosphoprotein P; those with insertions of 1G or 2G generate V and W proteins, respectively. Due to the translational frameshift, the amino acid sequences of V and W diverge downstream of the G insertion, while all three proteins have identical sequences upstream (white box). The divergent sequence at the C-terminus encodes binding sites for viral proteins, N and L (in P, grey color), a Cys and Pro-rich zinc finger domain (in V, vertical stripes), and the shortest protein (W) with the common sequence only (since the W reading frame generates a translational stop codon soon after the 2G insertion). The positions and lengths are not drawn to scale.

The viruses in this family generate IFN suppressor proteins by translation of alternative reading frames, produced by RNA editing of the P gene mRNA, likely to economize the use of the small RNA genome [91]. In this form of RNA editing, the viral RdRP inserts non-templated extra G residues at a certain frequency and at a specific site while transcribing the conserved P gene. While the unedited P mRNA codes for the phosphoprotein, a conserved subunit of the viral RdRP complex, insertion of 1 or 2 G nucleotides leads to translational frame shifts [92,93,94,95,96,97,98], and thus, in addition to the P protein, several IFN-antagonizing proteins are produced that differ in their sequence after the insertion. The convention is to designate the proteins V and W, respectively (Figure 3), of which V is the predominant and the more abundant IFN suppressor and hence the best studied [99,100].

**Figure 4:**
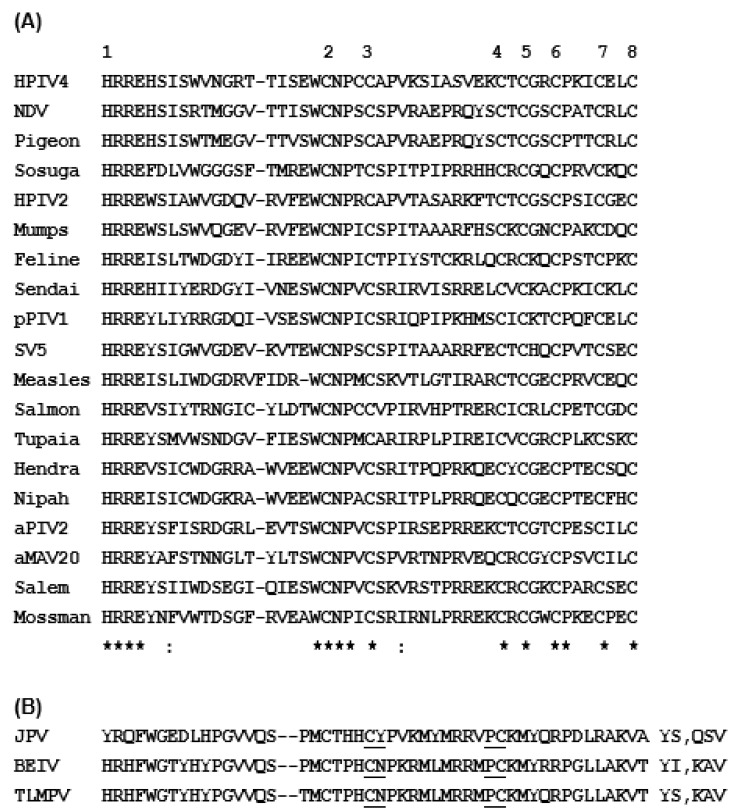
Conserved Cys-rich C-terminal domain (CTD) of the *Paramyxoviridae* V proteins. (**A**) Multiple alignment of the V protein primary structures was performed with Clustal Omega [104]; the invariant residues are asterisked, and one His and seven Cys residues that together coordinate two zinc ions to form a pair of zinc fingers are highlighted [100,101,102,103,105,106]. The numbers 1 through 8 on top indicate the eight residues of the zinc finger. Note the invariant Trp and the abundance of Pro residues, which may also be relevant to structure and function. The lack of helical structure in the zinc finger (Figure 5) is certainly in accord with the high concentration of Pro, a well-known helix breaker. The GenBenk accession numbers are (from top to bottom): AFB82774.1 (human parainfluenza virus 4), AHN08005.1 (Newcastle disease virus), ACK38030.1 (pigeon paramyxovirus 1), YP_009094030.1 (Sosuga virus), BAE00052.1 (human parainfluenza virus 2), AYI58047.1 (mumps virus), AFH55521.1 (feline morbillivirus), BAC79143.1 (Sendai virus), QCY53471.1 (porcine parainfluenzavirus 1), QCI44206.1 (SV5), ABB71650.1 (measles virus), AFF60403.1 (Pacific salmon paramyxovirus), NP_054692.1 (Tupaia paramyxovirus), NP_047108.1 (Hendra Henipavirus), NP_112023.1 (Nipah Henipavirus), ADK25231.1 (Avian parainfluenzavirus 2 or Avian metaavulavirus 2), YP_009553490.1 (Avian metaavulavirus 20), YP_009094333.1 (Salem virus), NP_958050.1 (Mossman virus). A combination of common and updated virus names were used to conserve space as well as to avoid identical abbreviations. Since these viruses are distributed over multiple genera, the zinc finger appears to be a general feature in this family, with exceptions shown below. (**B**) The three viruses of the *Paramyxoviridae* family were recently discovered in rodents and were placed in the genus Jeilongvirus [107]; these three (J-virus, JPV; Beilong virus, BEIV; Tailam virus, TLMPV) do make V proteins by RNA editing, but several zinc-coordinating residues in their CTD (see Figure 4) are naturally altered (shown in red color) so that the zinc finger cannot form [100]. All three are missing zinc-coordinating Cys residues #5, #6, #7, and #8 (also see Figure 5); JPV is additionally missing #1 i.e., His. To conserve space in the multiple alignment, I removed five residues from the underlined regions in each jeilongvirus sequence and display removed ones on the right; for example, in JPV, the dipeptide YS was present between C and Y as CYSY, and the tripeptide QSV was between P and C as PQSVC.

**Figure 5:**
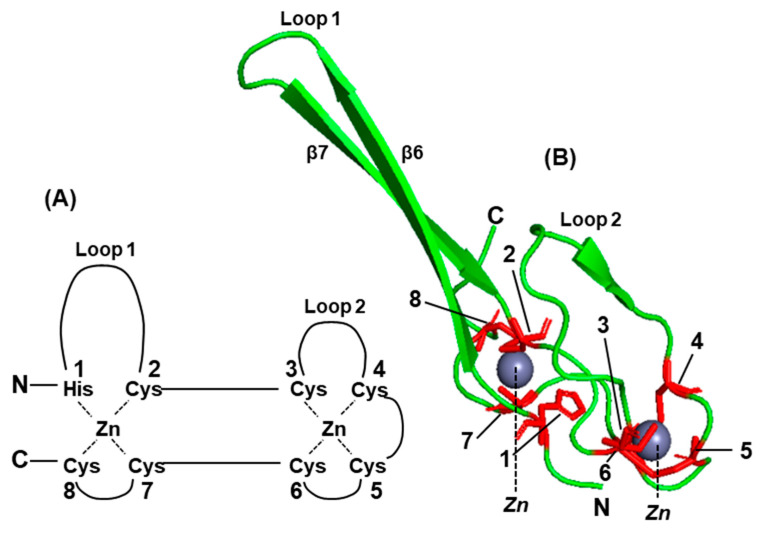
The structure of the zinc finger at the C-terminal domain (CTD) of the *Paramyxoviridae* V proteins. A consensus schematic (**A**) and the actual crystal structure of SV5 V protein zinc finger [105] in PyMol presentation (**B**) are shown. The eight residues participating in the coordination of zinc are numbered (and in Figure 4); the two intervening loops (large loop, L1; small loop, L2) and the zinc ions (gray spheres in panel (**B**); they are red spheres in Figure 6, panel (**A**)) are also indicated. The two beta-strands of the L1 loop (β6, β7) are marked. Note that in the jeilongviral V proteins critical zinc-coordinating residues (viz. residue #5, #6, #7, and #8 in all three viruses, and also residue #1 in JPV; Figure 4) were replaced by non-coordinating residues, and consequently, they cannot form a zinc finger.

The C-terminal domain (CTD) of the V protein is the most distinctive and folds into a zinc finger [92,100,101,102,103], which is important for STAT suppression (Figure 4 and Figure 5) [100]. The W reading frame encounters a new stop codon immediately after the 2G insertion, and as a result, W can be considered equivalent a deletion of the P sequence, containing only the N-terminal portion of P that is also common to P, V, and W (Figure 3). Due to their common as well as unique sequences, these three proteins exhibit a medley of interactions with the various members of the STAT family, which also varies from virus to virus.

##### The Nature of the V-Dependent E3 Ligase Complex and Its Role in STAT Regulation

Although the V proteins are involved in *Paramyxoviridae* IFN evasion, the molecular mechanisms of their action significantly differ between viral genera [100]. As a rule, V proteins of viruses belonging to the *Rubulavirus* genus (e.g., SV5, PIV2, mumps) target STAT1 or STAT2 for degradation by the UPS, whereas the *Henipavirus* and *Morbillivirus* (e.g., measles) V proteins bind to STAT1 and STAT2 and prevent their phosphorylation and nuclear import. Finally, unlike the STAT-degradative nonstructural proteins of the pneumoviruses (Section 2.3.3), the V protein is structural in nature, and consequently, STAT degradation in rubulavirus-infected cells can occur in the absence of viral protein synthesis [33]. In this section, our focus is on the *Rubulavirus* V proteins, but we will draw on their mechanistic differences from the non-degradative V proteins to further our understanding as needed.

**Figure 6:**
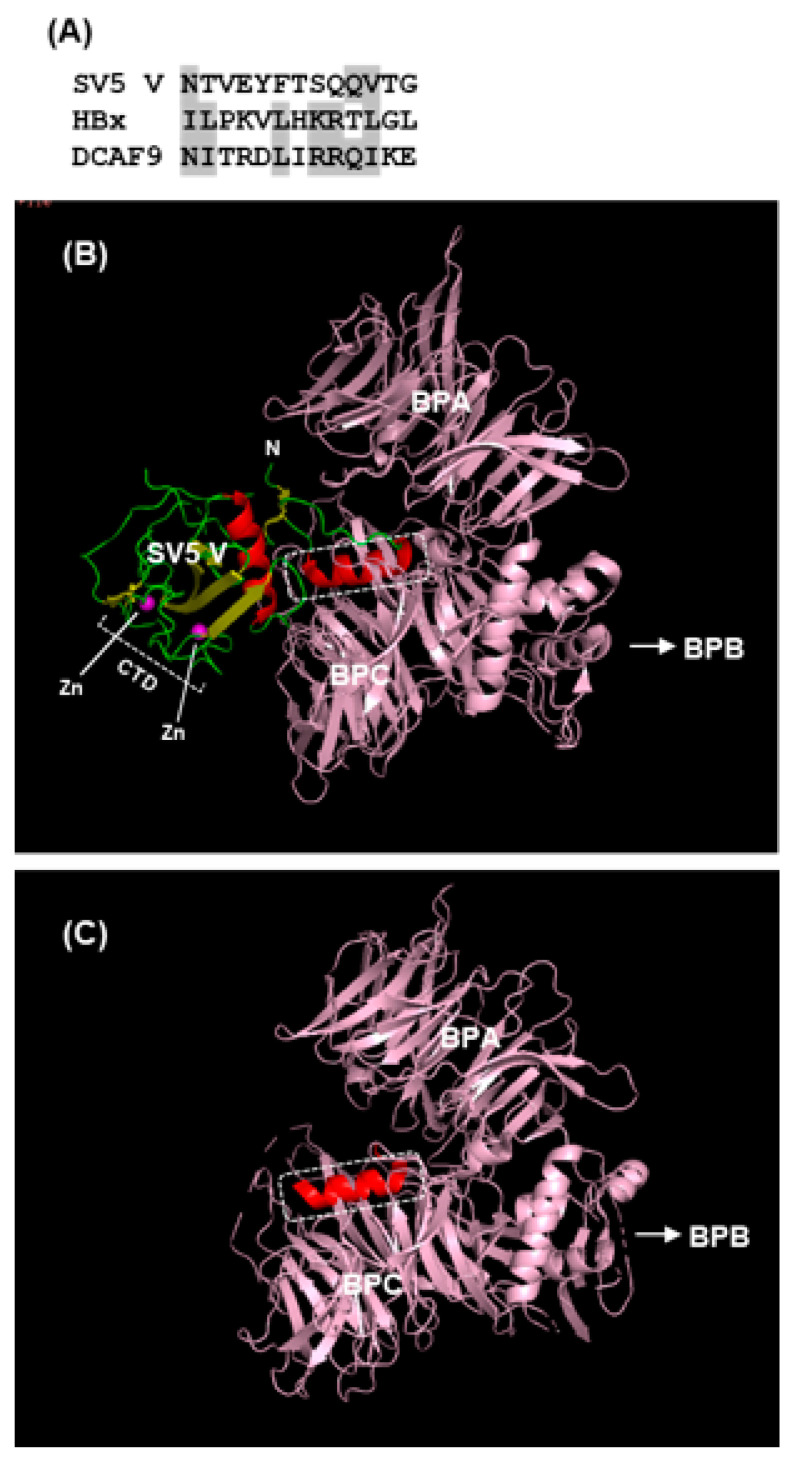
(**A**) Multiple alignment of the DDB1-interacting sequences in two viral proteins and a DCAF (DDB1–Cullin-interacting factor); the similar residues are highlighted. Portions of the cocrystal structure of DDB1 with (**B**) SV5 V protein and (**C**) the central H-box helical fragment of HBx (hepatitis B virus X protein). This is a PyMol presentation of PDB 2B5L and 3I7H, respectively [105,106]. Colors used are: V protein is green, except the helices, which are red; the H-box helix of HBx is also red; the zinc atoms are magenta; DDB1 is light pink. In the V protein, the N-terminus, the C-terminal domain (CTD), and the two zinc atoms in the CTD (as in Figure 5) are labeled. The boxed helices in both panels are structurally similar to each other, and both interact with DDB1 in a region between β-propellers A and C (BPA and BPC); the primary structures of these same helices are shown in panel (**A**). The β-propeller B is not shown to save space, but its direction is indicated with the arrowhead. Note that the DDB1 structure in panels (**A**,**B**) are essentially identical, although they may appear somewhat different because the images were rotated for the best visual alignment of the DDB1-interacting helices (boxed) in the two panels, without regard to DDB1 orientation.

Structure–function analysis has suggested that the Cys-rich C-terminal domain (CTD), particularly the zinc fingers (Figure 3 and Figure 4), of the V protein is required to stabilize the interactions of the V protein with DDB1, whereas direct binding occurs with the other regions (also see Figure 6) [105,106,107,108,109]. This is also supported by phylogenetic evidence; specifically, the three jeilongviruses that express V proteins incapable of zinc finger formation (Figure 4 and Figure 5) also fail to affect STAT1 or STAT2 [110].

The rest of the V protein, specifically the N-terminal helical extension and the globular core domain, interact directly with DDB1 (Figure 6). DDB1 is almost entirely made up of three intertwined β-propellers, designated BPA, BPB, BPC [105,106]. Crystal structures of apo-DDB1 and its complexes with several DDB1-binding proteins, including SV5-V, hepatitis B virus X, and cellular DCAF (DDB1–Cullin-interacting factor) proteins have been solved. They have revealed that a large pocket formed between BPA and BPC fully engulfs the SV5-V N-terminal helix (Figure 6) and functionally equivalent helices of all the others. Interestingly, the primary structures of these DDB1-binding helices are only distantly similar (Figure 6A). Although only DCAF9 sequence is shown here, a number of DCAF paralogs have similar DDB1-binding helices, leading to the notion that these viral proteins are mimics of cellular DCAFs in this regard [106], which was also suggested by studies of CMV STAT2 suppressor proteins, M27 and pUL145, as detailed in Section 2.3.5. It remains to be seen whether co-crystals of M27 and pUL145, when available, will also reveal similar helices.

DDB1 additionally forms a stable complex with Cul4A [111], suggesting a moonlighting role of the DNA-damage-binding DDB1 as a core subunit of the ubiquitin ligase complex, recently confirmed in a comprehensive analysis of the IFN-antagonistic mechanisms of diverse viruses, including herpes viruses and HIV (a retrovirus) [112]. In paramyxoviruses, in particular, the viral VADCs contain DDB1–Cul4A–Roc1 [62,105,113], representative of a type of Cullin–RING E3 Ub ligase (CRL). DDB1, as noted earlier, is a multifunctional protein, almost fully composed of β-strands that fold into three β-propeller domains, whereas Cul4 is essentially all α-helical.

The modular domain arrangement of these two proteins, in combination with Roc1 and virus-specific V proteins, likely offers the flexibility needed for the assembly and function of the VADCs [100,105,109,113], each of which functions as an E3 ligase and yet ubiquitylates specific STAT proteins, differentiating between STAT1 and STAT2. It is also intriguing that the V proteins of SV5 and PIV2 require both STAT1 and STAT2 to be able to degrade STAT1 and STAT2, respectively [34], and evidence suggests that V, STAT1, STAT2, and DDB1 may all assemble into one super-large VADC [62,113,114]. The rubulaviral V proteins actually display significant differences among themselves. First of all, their substrate choices differ: SV5 V protein degrades STAT1, HPIV2 V degrades STAT2, whereas MuV V degrades STAT1 and STAT3 [62,115]. Nevertheless, the VADCs assembled by all three contain DDB1, Cul4A, and Roc1 [115]. RNAi-mediated knockdown studies revealed that DDB1 is essential for the degradative actions of all three. However, Roc1 exhibits a more complex requirement; it is required for the STAT3-degradative activity of MuV VADC but is dispensable for STAT1- and STAT2-degradative activity of SV5 and HPIV2, respectively [115]. Finally, appropriately designed in vitro reactions, constituted with various combination of the VADC subunits along with STAT2, led to the conclusion that the V proteins have an intrinsic E3 ligase activity [62].

#### 2.3.3. Pneumoviridae

##### Viral Nonstructural Proteins, NS1 and NS2

Similar to the *Paramyxoviridae*, the viruses of the *Pneumoviridae* family belong to the *Mononegavirales* order [116] and possess a similar structure of the genome RNA and the ability to strongly suppress IFN-based innate immunity. However, the IFN-suppressor viral proteins are very different in sequence, and yet they target STAT proteins. Two major members of this cohort are respiratory syncytial virus (RSV) and the related mouse virus, murine pneumonia virus (MPV; previously, pneumonia virus of mice or PVM). The human RSV (HRSV) is the most significant cause of respiratory illness in infants and older individuals, especially those with compromised immunity, and no reliable vaccine or specific antiviral therapy is available yet [117].

In both RSV and MPV, type I IFN is suppressed primarily by two nonstructural proteins, so named due to their absence in the mature virus particles (i.e., virions). Designated 1 and 2 (NS1 and NS2), these proteins, unlike the V proteins of the paramyxoviruses, are expressed from independent genes [118,119,120,121]. Cumulative results from multiple investigators have shown that NS1 and NS2 share a few substrates, but each protein targets specific substrates as well [56,122,123,124,125,126,127,128]. In the majority of the literature, NS2 has been reported to degrade human STAT2 more consistently than NS1, but we subsequently showed that if higher amounts of NS1 are expressed by increasing the amount of transfected DNA, STAT2 degradation could be observed [127]. The individual contributions of the two NS proteins in the RSV-infected cells likely depend on the relative amounts of the steady-state levels that have not been accurately quantified. A major complexity of studying NS proteins stems from the fact that they form homomers as well as heteromers, i.e., NS1–NS1, NS2–NS2, and NS1–NS2, the exact stoichiometry being unknown [126,129]. Moreover, the NS proteins RSV and MPV have no similarity [126,129,130] between themselves or with any other protein in the sequence banks, making it difficult to recognize a domain. The C-terminal pentapeptide sequence (F/YDLNP), common between NS1 and NS2 of RSV, is not found in the MPV NS proteins. In the RSV NS proteins, this sequence was shown to be important for STAT2 degradation [126,129], but this has not been investigated further. The substrate repertoire [131] of the NS proteins of the two viruses are also remarkably different, although they share the ability to degrade the STAT2 of their natural hosts (human and mouse, respectively) through the use of UPS [130]. Finally, the NS proteins of PVM degrade mSTAT2 much more efficiently than RSV NS proteins degrade hSTAT2, when expressed in comparable amounts [130]. Thus, it should be realized that unlike the similarity among the members of the V protein family, such as the C-terminal zinc finger (Figure 5), the NS acronym only refers to their non-structural nature, not sequence similarity.

**Figure 7:**
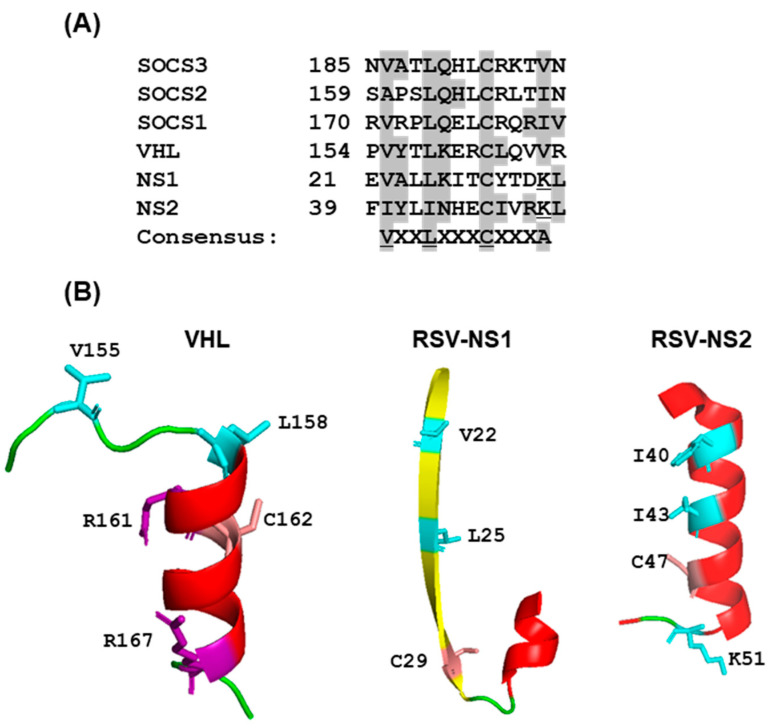
Putative BC box homology motif in NS proteins. (**A**) Distant homology of portions of NS1 and NS2 with known BC boxes, adopted from Elliott et al. 2007 [56] is shown by highlighted residues. The underlined K (Lys) is unique in NS1/1. (**B**) Actual crystal structure of this part of VHL (PDB 1VCB), RSV NS1 (PDB 5VJ2), and RSV NS2 (7LDK) showing lack of secondary structural similarity. The residues highlighted in panel A are also shown as side chains in PyMol presentation.

A previous study [56] showed that RSV NS1, expressed in cultured cells, can assemble the elongin–Cullin2-E3 ligase to degrade STAT2. In fact, NS1 was shown to contain regions that exhibit moderate sequence similarity with elongin C- and Cul2-binding motifs in VHL- and SOCS-family proteins (Figure 7A), although the role of these sequences in NS1 function was not investigated.

Further work revealed that Rbx1 is also involved in the formation of the NS1 ligase complex [56], which was confirmed by us. Subsequently, we noted similar VHL- and SOCS-family motifs in NS2 (Figure 7A), and also identified an E3 ligase complex assembled by NS2 that contained elongin C as well as Cul5 and Roc2 (instead of Cul2, Roc1 of the NS1 complex), thus documenting common as well as NS-specific subunits (our unpublished results). These authors suggested that NS1 has an innate E3 ligase activity, which is in contrast to the paramyxoviral V proteins (Section 2.3.2) that act as an adaptor to recruit cellular E3 ligases [56]; however, further studies are needed in this area to define the exact function of NS1 and NS2 in the VADC.

In two breakthrough studies, the crystal structures of RSV NS1 and NS2 were recently solved, but the structures appeared to be highly dissimilar [132,133]. When we compared the 3D structures of the postulated VHL-motif regions of NS proteins with that of the VHL [51], no structural similarity could be seen in either (Figure 7B); moreover, the presumptive consensus residues were not positioned in similar places. The role of the noted motifs (Figure 7A) in E3 ligase activity therefore awaits experimental validation. Our own search for potential BC Box Cys residues through mutagenesis of PVM NS proteins failed to identify a functional motif, as all Cys-Ala mutants were proficient in degrading mSTAT2 [130].

##### Search for NS-Interacting Domains of STAT Proteins

**Figure 8:**
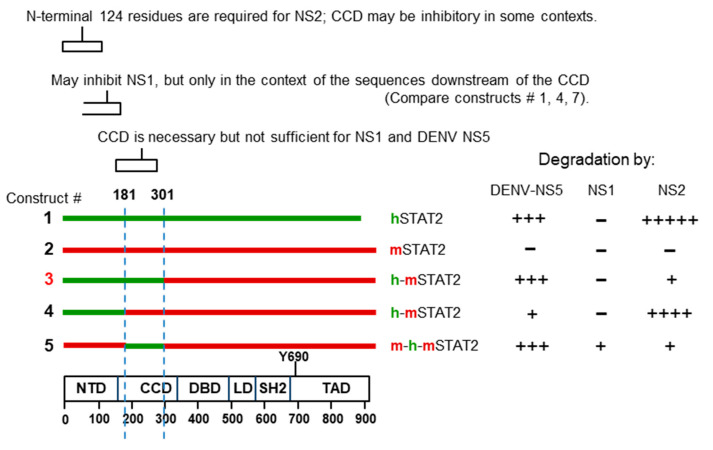
Distinctive actions of the two RSV NS proteins on human STAT2 (hSTAT2), mouse STAT2 (mSTAT2), and their chimera are compared with the activities of dengue virus NS5 protein on the same constructs [77]. Human sequences are colored green, and mouse sequences are red. The known motifs of STAT2 are marked in the schematic at the bottom, drawn to approximate scale by amino acid numbers. The roles of key sequence regions of the STAT constructs in NS1/2 and NS5 degradative functions are briefly described on the top. The degradative activity of equal amounts of the expressed proteins are qualitatively indicated by + and − signs; for example, NS2 is highly active on hSTAT2, but does not degrade mSTAT2 at all. See Section 2.3.3, article 2 for the details.

As mentioned earlier, the coiled-coil domain (CCD; 136–315) of hSTAT2 that connects the N-terminal domain (1–135 aa) to the DNA-binding domain (316–486) (Figure 2) was shown to be critical for its interaction with the DENV NS5 protein [75,77]. Similar to NS5, the RSV NS2 also degrades hSTAT2 but not mSTAT2 [123] and likewise may be a determining factor in the low pathology of RSV in mice, compared with the severe respiratory disease in human neonates. This knowledge of species-specificity and the availability of various hSTAT2–mSTAT2 domain chimera prompted us to attempt mapping of the STAT2 domain recognized by the RSV NS proteins. The Flag-tagged STAT proteins and their chimeric recombinants were expressed in human A549 cells by transfection, together with recombinant NS1 or NS2, and the STAT levels were determined by immunoblot with Flag antibody, followed by quantification of band intensities, normalized against actin (Supplementary Materials S1). In these experiments, equal but small amounts of NS1 and NS2 were expressed so that we could distinguish between the stronger degradative ability of NS2 and the weaker NS1. First of all, NS2 degraded human STAT2, but not mouse STAT2, confirming previous results [123,127]. The final results, provided as a schematic (Figure 8), show that the coiled-coil domain (CCD) does not ensure the degradation of hSTAT2 by NS2, unlike the case with DENV NS5 protein, since construct #5 with hSTAT2 CCD could not be degraded. Construct #3, which contains the N-terminal 300 residues of hSTAT2 that includes the CCD, was also resistant. Unexpectedly, loss of the CCD sequence from construct #3, resulting in construct #4, restores degradation. Thus, the CCD may actually have a negative effect on degradation by NS2 that counteracts the positive role of the N-terminal 120 residues.

It is obvious that the interactions between STAT and NS proteins are complex and probably involve multiple regions of the STAT sequence and contact points in the 3D structure. A similar conclusion could be reached by comparing between the various chimera of human STAT2 and human STAT1 (data not shown).

Although we used a limited number of constructs, these results point to a model in which the NS proteins straddle over different regions of STAT within a spatial distance, in addition to requiring specific amino acid residues to contact. A finer mapping, in conjunction with co-crystal structures of STAT and NS proteins in various combinations, should confirm these predictions while identifying the mutual contact residues.

#### 2.3.4. *Arteriviridae*

Similar to the *Flaviviridae*, the *Arteriviridae* family consists of viruses with nonsegmented positive-sense RNA that is translated into a polyprotein, which is processed into more than at least a dozen nonstructural proteins (NSP1 to NSP12). Recent studies have shown that NSP11 of PRRSV (porcine reproductive and respiratory syndrome virus), a prototype arterivirus, inhibits IFN signaling by promoting UPS-mediated STAT2 degradation, while STAT1 was not affected [134]. STAT2 levels could be restored in the presence of proteasomal inhibitors MG132 and lactacystin; additionally, ubiquitylated STAT2 could be demonstrated. Structure of apo-crystals of NSP11 showed two compact domains, designated N-terminal and C-terminal domains (NTD and CTD, respectively), dominated by β-strands [135]. Biochemical structure–function analysis, in the absence of a NSP11–STAT2 co-crystal structure, suggested that the NTD of NSP11 interacts with the NTD and CCD of STAT2. Interestingly, NSP11 is also an endoribonuclease that cleaves both single- and double-stranded RNA, but its role in PRRSV or its relationship, if any, with the STAT2 degradation function is unclear. The sequence or 3D structure of NSP11 shows no discernable similarity with those of the other STAT2-degradative viral proteins such as flaviviral NS5, paramyxoviral V, or pneumoviral NS1/2 proteins (our unpublished observation). Taken together, the combined evidence suggests that the viral nonstructural proteins use disparate interacting surfaces to achieve STAT2 degradation.

#### 2.3.5. *Herpesviridae*

The *Herpesviridae* family of double-stranded DNA viruses comprise several clinically important human pathogens, including several common ones, such as the genital herpes simplex virus (HSV), varicella zoster virus (causing chickenpox and shingles), Epstein–Barr virus (implicated in several diseases), and human cytomegalovirus. All are widespread among humans and often reside in a latent state until reactivation occurs with resultant disease.

The cytomegalovirus (CMV), representing the *Betaherpesvirinae* subfamily, is in fact one of the first viruses for which proteasomal STAT2 degradation was reported [24,135]. The prototype betaherpesviruses are the human CMV (HCMV) and mouse CMV (MCMV) that contain collinear genomes and exhibit similar pathology. In MCMV, the viral protein M27 (UniProtKB: A2Q6L2) degrades STAT2 using UPS in a DDB1–Cul4A/B-Roc1-based mechanism [25,112,136,137]. Surprisingly, the corresponding homolog in HCMV, named UL27 (UniProtKB: P16763), was found to be devoid of this activity, even though it shared ~24% amino acid identity with the murine sequence. Nonetheless, HCMV also degrades STAT2 to evade host immunity, which was discovered to be due to the viral gene UL145 [138]. The protein product, designated pUL145, showed no similarity with M27. Remarkably, ribosome profiling studies revealed that the UL145 gene generated two pUL145 isoforms—the full-length 130 aa long protein and a shorter, 113 aa long isoform—resulting from initiation at a downstream Met. In the HMCV-infected fibroblasts, the short form is actually the main product of the UL145 gene, as it is expressed earlier and more abundantly. Nevertheless, both isoforms of pUL145 recruit cellular DDB1–ubiquitin ligase complexes, using a motif that showed significant sequence similarity with the H-box motif of cellular DDB1 Cullin-associated factors or DCAFs, which serve as the DDB1-binding interface. Site-directed mutagenesis of the pUL145 residues, conserved within this DCAF-like motif, indeed resulted in loss of DDB1-binding [138]. Although the 3D structure of M27 or pUL145 has not been reported yet, recall that such interactions between the DDB1 and the H-box motifs in SV5 V and hepatitis B virus X proteins were revealed in the co-crystal structures (Figure 6), suggesting commonality of mechanism at the molecular level among diverse viruses. Finally, similar to the DDB1-binding M27, pUL145 also uses the CUL4A/B-Roc1 E3 ligase to degrade STAT2 by cellular UPS [139,140]. The species-specific difference of the STAT2 suppressor genes of CMV is in contrast with the V proteins of multiple rubulaviruses that infect different hosts but still display significant sequence homology and assemble a functional STAT2-degradative VPAC (Section 2.3.2, article 2).

HSV2 (Table 1), another member of this family, uses the viral protein ICP22 (infected-cell protein 22) to degrade not only STAT2 but also STAT1 and IRF9, i.e., all three proteins of ISGF3, thus destroying the entire complex. Again, the degradation is mediated by cellular UPS, as evidenced by ubiquitylation of the substrates and inhibition of degradation by bortzeomib, a proteasome inhibitor [61]. In this mechanism, ICP22 likely functions as an E3 ligase. A detailed mechanism of its interactions with the substrates should yield interesting insights into how ICP22 targets all three proteins (see Section 2.3.5).

## 3. Efficiency of Viral Suppression of IFN

A legitimate query of both academic and clinical interest is the extent to which viral suppression of STAT2 translates into IFN evasion. Directed studies and anecdotal evidence, briefly presented here, suggest that the effect may be an incomplete one. Moreover, suppression by different viruses vary in efficiency. In a 2003 report [141], the authors engineered established cell lines to express the V protein of SV5 (which suppresses IFN by targeting STAT1, as mentioned before; Section 2.3.2) and confirmed that the engineered cells no longer respond to IFN. It was then found that RSV yield was much higher and that the viral plaques were larger in these cells than in the original ones. Similar improvement in viral replication was observed for many other viruses [141] that are not discussed here to conserve space. The simplest explanation of this result is that suppression of IFN by SV5 V protein is more robust that by RSV NS proteins. More recently, wild-type and STAT2-KO mice were shown to exhibit fundamentally different susceptibilities to mouse CMV (MCMV) [142]. It is to be recalled that MCMV suppresses STAT2 by the use of its M27 protein (Section 2.3.5). In contrast with the wild-type animal, MCMV replication in the KO mice was found to be elevated in all assessed organs, resulting in mortality within the first week of infection [142]. Thus, even though M27 degrades STAT2, the process was likely incomplete, and the remaining STAT2 prevented overt pathology and mortality upon MCMV infection. These results not only underscore the multiple considerations that should be taken into account in understanding host–virus immune interactions but also illustrate the evolutionary balance between virus growth and host survival.

## 4. Summary and Directions for Future Research

**Figure 9:**
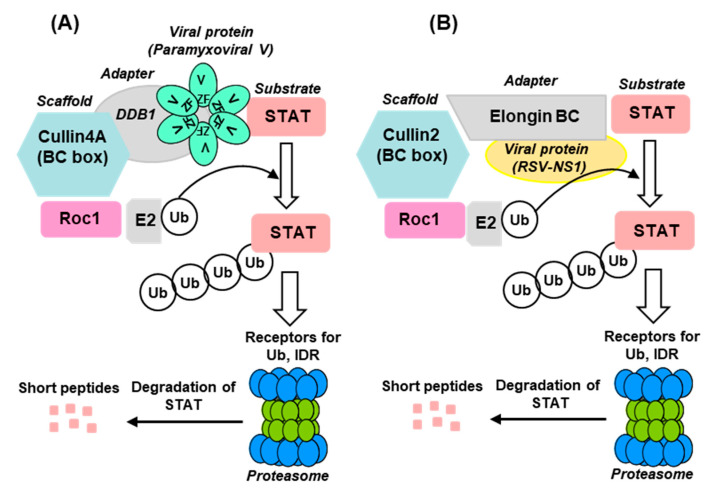
Working model of representative virally assembled proteasome complexes (VADCs) of the V protein (**A**) and RSV NS proteins (**B**). The various subunits of the VADC are indicated, which include the CRL components as shown, i.e., CUL4A–Roc1 E3 ligase and DDB1 as adaptor protein in the V protein VADC (**A**), and CUL2–Roc1 E3 ligase (in HEp-2 cells) and elongin BC in the RSV NS1-based VADC (**B**). The viral protein serves as the STAT2-recognition protein. However, as described in Section 2.3.2, V proteins also have an intrinsic E3 ligase activity, thus serving both as a substrate recognition protein and an E3 ligase. It is unclear whether the viral NS proteins act in both capacities since their E3 ligase activity remains to be characterized in greater detail. Among the cytomegaloviruses (not shown here, but detailed in Section 2.3.5), the M27 protein of mouse CMV and the nonhomologous pUL145 protein of human CMV both use DDB1 as adapter and the CUL4A/B-Roc1 E3 ligase for STAT2 ubiquitylation and eventual degradation, which is reminiscent of the V proteins (**Panel A**). In all cases, the ultimate result is ubiquitylation of STAT2, followed by recognition of an intrinsically disordered region (IDR) of Ubq–STAT2 by a receptor at the proteasome entry tunnel, and finally, degradation. See Section 2.1 and Section 2.2 for details.

The hijacking of the cellular UPS to degrade STAT2 appears to be a common immune evasion strategy adopted by the viruses, regardless of the RNA or DNA nature of their genome. The current state of our knowledge of the viral mechanism of STAT2 degradation is schematically depicted here (Figure 1 and Figure 9), using the paramyxoviral and pneumoviral VADCs as main representatives (Section 2.3.2 and Section 2.3.3). The general pattern that emerges is that the viral proteins function as the STAT2-recognition subunit that recruits STAT2 to the specific CRL (E3 ligase) that consists of various combinations of Cullin (e.g., Cul1, Cul2, Cul4/5), RING-finger protein (e.g., Roc1/Rbx1, Roc2/Rbx2) and adapter protein (DDB1, elongin BC) (Figure 1 and Figure 9). Even though the mechanisms are incomplete in specific molecular details, the diagrams should serve as working models for further studies.

It is relevant to mention here that regulation of STAT2 levels also occurs in the uninfected cell, at least one of which has been documented to utilize the CRL-dependent UPS system [142]. Briefly, in this particular mechanism, STAT2 is first phosphorylated by GSK3β (glycogen synthase kinase 3, beta isoform), and the resultant phosphor-STAT2 binds the cellular F-box protein FBXW7 (See Section 2.2). As alluded to earlier, the F-box proteins represent one class of CRL, in which the F-box protein acts as the substrate-recognition protein, Skp1 protein acts as the cognate adapter, and they use Cul7–Roc1 E3 ligase to ubiquitylate the substrate [59]. Several F-box proteins, including FBXW7, contain various repeats, such as the WD40 repeat that has a beta-propeller structure [143,144]. Studies of Lee et al. [143] revealed that specific phosphorylated residues of STAT2 make contacts with the WD40 domain of FBXW7, which prepares STAT2 for ubiquitylation and eventual UPS-mediated degradation. Clearly, when the viral proteins take over the substrate-recognition function in the infected cell, they interact with a different adapter and enlist a different CRL to ubiquitylate the same substrate, i.e., STAT2 in this case. This is an example of functional similarity of diverse CRLs, a hallmark of E3 ligases, as discussed further in Section 4.1.

In what follows, a few unresolved aspects of these mechanisms are summarized, and possible future directions of research are outlined.

### 4.1. Diversity, Multiplicity, and Specificity

Cellular E3 ligases are enormously diverse, and the CRL family is no exception. Examples abound where the same substrate, for example STAT2, is ubiquitylated by different CRLs, such as Cul4A–Roc1 E3 ligase recruited by DDB1-using morbillivirus V proteins and Cul2–Roc1 E3 ligase recruited by elongin BC-using RSV NS protein (Figure 1 and Figure 9). Reciprocally, the same viral protein often promotes ubiquitylation of multiple substrates, such as the RSV NS1 promoting UPS-mediated degradation of RIG-I, TRAF3, IKKε, and IRF3, although it is currently unknown whether the same CRL is used for all these substrates.

Even a cursory look at the CRL–viral protein combination reveals the multiplicity of pairing. For example, M27 and pUL145, two highly dissimilar proteins, both use CRL4A/CRL4B that have identical adapter protein (DDB1) and normally employ DCAF as the substrate receptor in the uninfected cell. As previously suggested [112], the choice of Cul4A versus Cul4B depends on their relative affinity for DDB1 as well as the affinity of the viral protein for DDB1. In other words, the stability of the holocomplex (Figure 1 and Figure 9) derives from the sum of the strengths of interaction between each neighboring pair of proteins.

Traditionally, direct analysis of the composition of the VADCs uses affinity-purified complex—for example, pulled down by tags attached to recombinantly expressed viral protein—followed by immunoblot that led to the identification of the subunits as presented here (Figure 1 and Figure 9). However, unbiased staining (e.g., silver-staining) of all proteins in the complex, generally revealed a plethora of proteins [62,145] (our unpublished results), the identities of many of which remain unknown. The V-dependent complexes of SV5 and MuV, for example, contained Cullins 1 and 2, in addition to Cul4A, although it is not known whether Cul1 and Cul2 are functionally relevant (Christina Ulane, PhD thesis, and Curt Horvath, personal communication). Nonetheless, it is possible that Cul1/2 may play some role, if minor, since the high structural similarity of the Cullins sometimes translates into overlapping functions [146,147], such as their interaction with Roc 1 (Figure 1). Alternatively, one Cullin may bind to Roc1 more efficiently than the other Cullins, and may eventually win the competition. In this scenario, a specific Cullin is preferred because its association is strengthened by the other interactions in the VADC, reminiscent of the preference of M27 for Cul4B, mentioned above. It is also possible that the VADC, at the earlier steps of assembly, is a metastable complex that switches its Cullin homologs, as has been proposed recently for DCAF switching [148].

In summary, multiple viral proteins, serving as E3 ligases, recruit different homologs of the host proteins as accessories to form the STAT2-degradative holocomplex. As there are dozens of E2 enzymes and a few hundred E3 ligases in a given cell to choose from, these “accessory” proteins serve to integrate and converge the viral diversity into the conserved STAT2 and the proteasomes. We propose the name “isosubstrate enzymes” for such a family of dissimilar “enzymes” that use the same substrate. The terms of the mutual co-evolution of the viral suppressors and their cognate host proteins have been articulated elsewhere [149].

The other side of the “isosubstrate enzymes” is the multiplicity of substrates targeted by the same viral protein. Specifically, several viral degraders—albeit not all—ubiquitylate and degrade a few other proteins in addition to STAT2. For instance, in contrast with the NS2 protein of RSV, the NS1 protein is relatively promiscuous, as it targets at least five substrates, viz. RIG-I, TRAF3, IKKe, IRF3, IRF7 [126,127]. How does NS1 degrade five proteins that are very different in amino acid sequence, but is still specific enough to spare thousands of other cellular proteins? This “promiscuity versus specificity” question is a challenging one, but we offer the following hypothesis, based on the functional commonality among these substrates, as they all belong to the IFN pathway. Our working hypothesis is that they first undergo an IFN-triggered post-translational modification. We like to speculate that this modification is conjugation to ISG15 (IFN-stimulated gene 15), a small Ubq-like (UBL) protein, in a process known as ISGylation [150]. ISG gene expression and ISGyation of selective proteins is induced by a variety of stress signals, including IFN, virus infection, and plasmid transfection. Specifically, we propose that infection by RSV or transfection by NS-expressing plasmids induces ISGylation, and the ISG moiety acts as the tag for recognition by NS1. Several IFN pathway proteins, including RIG-I, MDA5, IRF3, JAK1, and STAT1, are in fact known to be modified by ISG15, and new ones are being discovered routinely [150]. Unlike K48 ubiquitylation, ISGylation of proteins generally does not target them for proteasomal degradation; thus, it is believed to regulate protein function and promote protein–protein interaction. As ISGylation is a reversible process, several viruses, including SARS-CoV-2, encode enzymatic activities that catalyze deISGylation, i.e., removal of the ISG groups, causing inactivation of the immune protein and evasion of IFN [151,152], which underscores its importance in virus infection. We propose that degradation of the ISGylated substrate is an additional mechanism used by viral proteins that employ UPS. These viral proteins, exemplified by RSV NS1, thus recognize the same ISGylation that promotes IFN function. In other words, IFN-induced ISGylation not only serves as a call for action to NS1 but also provides a specific barcode for destruction of these same proteins. This would limit unnecessary use of NS1 to degrade IFN-irrelevant cellular proteins while allowing it to strongly antagonize IFN response by targeting multiple players of the IFN pathway. Nevertheless, additional specificity is likely determined by various subunits of the VADC, such as the adapter elongin BC or the cognate Cullin–Roc1 pair, so that not all ISGylated proteins are recruited to the VADC.

### 4.2. Directions for Future Research

This section builds on what has been presented so far and suggests a few areas of new research that can further our knowledge of how viruses degrade STAT2.

(i) First and foremost is to identify the E2s and E3s of a given VADC, for which established analytic procedures should suffice. It will likely be necessary to prevent STAT2 degradation in the complex using suitable proteasome inhibitors, and the ubiquitin chain needs to be trimmed to a uniform homogenous length. The next step is to determine the three-dimensional structure of the holocomplex.

In addition to the usual difficulties of recombinant expression and purification of the proteins, the crystallization of their complex in vitro, followed by high-resolution diffraction, poses significant hurdles. However, it may not be impossible, as structures of large multiprotein complexes have been determined by several investigators in the past, notable among which are ribosomes [153,154,155,156] and the eukaryotic DNA-dependent RNA polymerase II [157].

(ii) Comparative analysis of the VADCs of two viruses of the same family, such as the NS1/NS2 complexes of human RSV and mouse MPV should illuminate the mechanism of each as well as identify the interactive side residues, and hence their co-evolution. Such studies can also be extended to the differential susceptibility of mouse and human STAT2 and between STAT1 and STAT2.

Much can also be learned from the differential action of the same viral protein on different members of the STAT family from the same species. Perhaps the most detailed analysis of the STAT-preferences of the P, V, and W proteins were performed with the Nipah virus (NiV) proteins by several investigators, which revealed a complex pattern, reviewed in a recent publication [158]. The findings are summarized here in brief only to illustrate this complexity, but the mechanisms vary among the paramyxoviruses, and therefore, the NiV results may not be generalized for all. In brief, NiV P, V, and W bind STAT1 as well as STAT4 using the common N-terminal sequence, but none of them bind STAT3 or STAT6. The V protein, which has the zinc finger domain at the unique C-terminus, additionally binds STAT2 and STAT5. The interactions with STAT1 and STAT2 block their tyrosine phosphorylation and IFN signaling. Overall, the Nipah viral proteins inhibit the function of several STAT proteins by sequestration without degrading them. Likewise, the measles virus V protein inhibits phosphorylation of both STAT1 and STAT2 by interacting with JAK [159], to name a few. The STAT preferences of the pneumoviral NS1 and NS2 have been already described (Section 2.3.3). Although it will entail a formidable amount of work, studies of such differential action should shed light on the interacting domains of the viral protein in question and the STATs.

On a related note, in the dual-STAT VADC, assembled by the V proteins of SV5 and PIV2 [113,114], how STAT1 is selected for degradation while STAT2 in the same complex is spared remains a mystery.

(iii) It is unknown whether in mixed expression, NS1 and NS2 are incorporated in the same VADC, and if so, how the structure of that complex differs from the individual NS1 complex and NS2 complex.

(iv) The ISGylation hypothesis proposed earlier (Section 4.2) is easily testable. If it is indeed correct, NS1 would not degrade its substrates under ISG15-deficient conditions, which can be tested in deficient cell lines and in knockout mouse that are also available [158], but NS2 may still work. In these cells, transfection with recombinant wild-type ISG15 will restore NS1 function, whereas the conjugation-defective C-terminal LRLRAA mutant ISG15 [160] will not.

(v) The putative E3 domains (BC box motifs) (Figure 7A, Section 2.3.3) in NS1/2 are awaiting experimental verification. The obvious first experiment would be to mutagenize it and test the functionality of the mutant. It is to be mentioned that this was recently attempted by Straub et al. [161], who created a recombinant RSV in which all three consensus amino acids of NS1 were mutated Ala, from VALLKITC to AALAKITA. Unexpectedly, the mutant NS1 in the infected cells was highly unstable and located to cytoplasmic bodies, even though the first amino acids were conservative hydrophobic replacements. Moreover, the mutant virus exhibited poor growth properties, comparable with that of the ΔNS1 virus [161]. It remained untested whether this mutant NS1 could be expressed recombinantly from a plasmid in uninfected cells. In NS2, we previously mutated the invariant Cys to Ala and found that the recombinant protein was stable and active in degrading STAT2 [129]; however, this could be because the Cys residue (alone) may not be absolutely essential for function since it is naturally replaced by Ala in the HIV Vif protein SOCS Box, which is functional and actually binds elongin C [56,162,163]. Thus, the NS1 and NS2 BC boxes may be functional as well, but this remains to be tested.

(vi) On a side note, a recent study of DENV type 3 NS5 crystal structure revealed that it can form two different global conformations, one of which resembles that of the Japanese encephalitis virus (JEV) NS5 [164]. Whether the bi-morphic nature and the interconversion of the structures are relevant for the IFN suppression function and STAT2 degradation activity of NS5 is a stimulating enquiry for future investigations and can shed light on the structure–function relationship of this multifunctional viral protein that is involved in both RNA and protein metabolism.

(vii) Therapeutic potential: In principal, any drug that enhances antiviral immunity or inhibits the viral suppression of immunity can act as an antiviral. In reality, such drugs also need to be specific since cellular immune functions may also have housekeeping roles. One class of inhibitors is based on the fact that Cullins are activated by conjugation of the ubiquitin-like protein NEDD8 (neural-precursor-cell-expressed developmentally down-regulated 8), through a process known as NEDDylation [165,166]. Two small-molecule inhibitors of NEDDylation, viz. MLN4924 (pevonedistat) and TAS4464, were shown to inactivate CRL complexes and suppress tumor growth [167]. Phase III clinical trials of pevonedistat in the treatment of acute myeloid leukemia (ClinicalTrials.gov Identifier: NCT04090736) are currently in progress [168]. Since many viruses suppress cellular immune response by using CRLs (Table 1 and Figure 1), both MLN4924 and TAS4464 were tested for their ability inhibit virus growth. Indeed, nanomolar concentrations of both drugs inhibited multiple viruses, including but not limited to HSV2, HSV1, and CMV [168,169]. It remains to be seen whether either drug is approved as a broad-spectrum antiviral in the clinic in due course.

## Figures and Tables

**Table 1:** Viral subversion of STAT2.

Virus Family/Name of Virus	Viral Protein(s) Promoting Degradation	Host Factors Needed for STAT2 Degradation
*Flaviviridae* (Section 2.3.1)		
Dengue	NS5	UPS, UBR4
Zika	NS5	UPS
*Paramyxoviridae* (Section 2.3.2)		
PIV2	V	UPS (DDB1, STAT1)
Mumps	V	
*Pneumoviridae* (Section 2.3.3)		
RSV	NS1, NS2	UPS
MPV	NS1, NS2	UPS
*Arteriviridae* (Section 2.3.4)		
PRRSV	NS11	
*Herpesviridae* (Section 2.3.5)		
HSV2	ICP22	UPS
HCMV	pUL145	UPS
MCMV	M27	UPS

Abbreviations: HSV2, herpes simplex virus 2; CMV, cytomegalovirus (H = human; M = mouse); PIV2, parainfluenzavirus 2; RSV, respiratory syncytial virus; MPV, mouse pneumonia virus (previously PVM, pneumonia virus of mice); NS, nonstructural; ICP, infected cell protein; DDB, double-stranded DNA-binding protein; UPS, ubiquitin–proteasome system; UBR4, E3 ubiquitin-protein ligase 4; STAT, signal transducer and activator of transcription. Note that the cellular UPS is overwhelmingly used by multiple viral proteins to degrade STAT2. Several viral proteins listed here possess E3 ligase activity of their own (e.g., ICP22), which is not indicated here to conserve space, but described later in individual virus sections.

## Supplementary Materials

The following supporting information can be downloaded at: https://www.mdpi.com/article/10.3390/ijms23010489/s1.
