# Supplementary material for: Mechanisms of Viral Degradation of Cellular Signal Transducer and Activator of Transcription 2
Source: Int J Mol Sci. 2022 Jan 1;23(1):489. doi: 10.3390/ijms23010489 (PMC8745392; doi:10.3390/ijms23010489)
Supplement: Supplementary file 1 [file ijms-23-00489-s001.zip › ijms-1480721-supplementary.pdf]

Supplementary Material

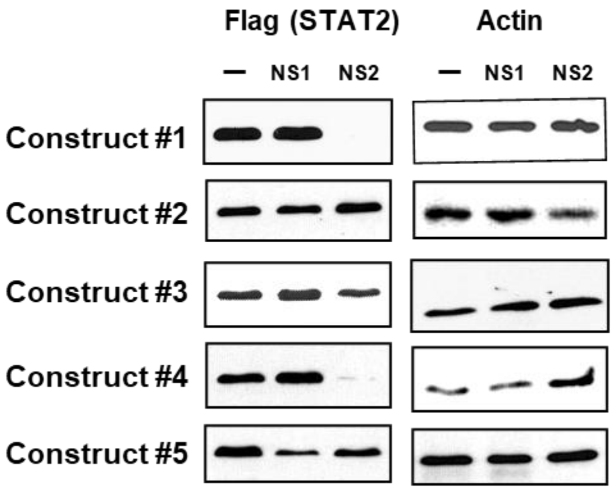

Figure legend. Immunoblot, showing various FLAG-tagged STAT2 proteins (Left panel) and control actin (Right panel), detected by antibody to FLAG and actin, respectively. The Flag-tagged STAT2 proteins were generated by transient transfection of appropriate plasmid constructs, described in detail in Figure 8. The NS proteins were also expressed from co-transfected recombinant plasmids. In brief, construct #1 expressed hSTAT2; construct #2, mSTAT2; construct #3-5, various chimera between the two, designed to map the domain(s) required for degradation by respiratory syncytial virus (RSV) NS1 or NS2. Total cell lysates were analyzed by immunoblot (Western blot) to detect the amounts of undegraded (remaining) STAT2, as shown. Same relative amounts of samples were loaded in the actin panel, to normalize the STAT2 band intensities. The plotted results are presented in Figure 8, where the corresponding results with the same constructs were obtained from Ashour et al (2010), cited below.

Reference:

Ashour, J.; Morrison, J.; Laurent-Rolle M.; Belicha-Villanueva, A.; Plumlee C.R.; Bernal-Rubio, D.; Williams, K.L.; Harris, E.; Fernandez-Sesma, A.; Schindler, C.; García-Sastre, A. Mouse STAT2 restricts early dengue virus replication. *Cell Host Microbe* **2010,** *8*, 410–421. DOI: 10.1016/j.chom.2010.10.007
